# Association of Individual and Neighborhood Characteristics to Problematic Internet Use among Youths and Adolescents: Evidence from Vietnam

**DOI:** 10.3390/ijerph20032090

**Published:** 2023-01-23

**Authors:** Thao Thi Phuong Nguyen, Ha Ngoc Do, Thao Bich Thi Vu, Khanh Long Vu, Hiep Duy Nguyen, Dung Tuan Nguyen, Hoang Minh Do, Nga Thi Thu Nguyen, Ly Thi Bac La, Linh Phuong Doan, Tham Thi Nguyen, Huong Lan Thi Nguyen, Hoa Thi Do, Carl A. Latkin, Cyrus S. H. Ho, Roger C. M. Ho

**Affiliations:** 1 Institute for Global Health Innovations, Duy Tan University, Da Nang 550000, Vietnam; 2 Faculty of Medicine, Duy Tan University, Da Nang 550000, Vietnam; 3 Youth Research Institute, Ho Chi Minh Communist Youth Union, Hanoi 100000, Vietnam; 4 Vietnam Youth Academy, Hanoi 100000, Vietnam; 5 Department of Research on Youth’s Organisations and Youth Campaign, Youth Research Institute, Ho Chi Minh Communist Youth Union, Hanoi 100000, Vietnam; 6 Department of Research on Children’s Issues, Youth Research Institute, Ho Chi Minh Communist Youth Union, Hanoi 100000, Vietnam; 7 Department of Research on Youth and Legal Issues, Youth Research Institute, Ho Chi Minh Communist Youth Union, Hanoi 100000, Vietnam; 8 Department of Research on Youth Culture and Lifestyle, Youth Research Institute, Ho Chi Minh Communist Youth Union, Hanoi 100000, Vietnam; 9 Faculty of Social Sciences and Humanities, Hanoi Metropolitan University, Hanoi 100000, Vietnam; 10 Faculty of Preschool Education, Hanoi National University of Education, Hanoi 100000, Vietnam; 11 Institute of Health Economics and Technology, Hanoi 100000, Vietnam; 12 Bloomberg School of Public Health, Johns Hopkins University, Baltimore, MD 21205, USA; 13 Department of Psychological Medicine, Yong Loo Lin School of Medicine, National University of Singapore, Singapore 119228, Singapore; 14 Institute for Health Innovation and Technology (iHealthtech), National University of Singapore, Singapore 119077, Singapore

**Keywords:** internet addiction, problematic internet use, adolescence, youth, Vietnam

## Abstract

Introduction: This study aimed to determine latent profiles from the Problematic Internet Use Questionnaire Short Form-6 (PIUQ-SF-6) score of Vietnamese youths and adolescents, which supports the diagnosis of problematic internet use among a large sample size. Moreover, it also explored factors that affect each latent profile of the PIUQ-SF-6 score among participants. Methods: A sample of 1477 Vietnamese people, aged 14 to 24, across five provinces participated in the study. Multinomial logistic regression determined factors related to the levels of the Problematic Internet Use Questionnaire Short Form-6 (PIUQ-SF-6) after using latent profile analysis. Results: Participants were divided into three profiles, including those at low, moderate, and high risk of internet addiction. The high-risk latent profile was obtained for 23.1% of adolescents, and the remaining percentages were, respectively, 40.2% and 36.7% of adolescents belonging to the moderate and low-risk groups. Moreover, factors including age, living alone, high Kessler psychological distress scale, excessive time on the internet, living in central cities, and high neighborhood disorder scores were found to be related to moderate- and high-risk internet addiction profiles. Conclusions: Factors analyzed according to individual and social characteristics further explore the reasons underlying increasing internet addiction among Vietnamese youths and inform early interventions.

## 1. Introduction

In recent decades, internet usage has become increasingly popular and has become an indispensable part of modern life worldwide. It is utilized as a convenient, cost-efficient means of communication and connection among the general population, especially among youths and adolescents [1]. However, internet addiction or excessive internet use may lead to negative physical, behavioral, and psychological consequences [2,3,4]. Internet addiction comes in various forms, including gaming addiction, pornography addiction, social media addiction, or virtual reality addiction [5,6,7,8,9]. Low self-esteem, disconnection from reality, short attention span, and reduced creativity are among the most common symptoms of internet addiction [10]. It is also the underlying cause of various health problems such as functional impairments in daily activities or concomitant mental disorders including attention deficit disorder, depression, and substance abuse [11,12,13,14]. Ongoing global research has been conducted on increasing reports of prevalence. One previous systematic review reported that the prevalence of problematic internet use ranged from 0.8% to 26.7% [15]. Another review of internet addiction in 31 nations determined that the highest prevalence of internet addiction was in the Middle East (10.9%) and the lowest in Northern and Western Europe (2.6%) [16]. In light of its substantial impacts, internet addiction has been well studied as a serious public health issue [14,17] and regarded as a priority in the digital era [18].

Youths and adolescents are among the most vulnerable populations to problematic Internet use. According to a national survey in Germany and a study in Siberia, the highest prevalence of internet addiction was found among people under the age of 25, at 5.2% and 7.2%, respectively [19,20]. In telephone surveys across worldwide populations, such as the United States (2006) [21], Germany (2014) [22], and Norway (2009) [23], findings revealed that most participants in the 14- to 24-year-old age group were at a higher risk of online addiction than other groups. In Vietnam, although previous studies had shown the prevalence of internet addiction and its related factors among teenagers and adolescents, the majority of these were only implemented in small areas (such as a province/city or a school/college/university). Therefore, there is a lack of large-scale and representative studies on this issue. Nevertheless, among the available literature, the prevalence of internet addiction among Vietnamese youths and adolescents was relatively high at 20−25% [24,25,26]. These studies indicated a relationship between individual factors (such as family situation, life satisfaction, self-esteem, depression, and anxiety) and problematic internet use among Vietnamese youth and adolescents [24]. Moreover, the consequences of internet addiction, such as insomnia, reducing the quality of life, or physical impairment [25,27,28] were also explored.

Most previous Vietnam studies used Young’s Internet Addiction Test (IAT) by Diagnostic and Statistical Manual of Mental Disorders (DSM) IV or V criteria for assessing internet usage, but its limitations were also mentioned as validating [25,26,27,29]. However, evaluating the severity of Internet addiction and duration of illness were also limitations of this instrument [30]. Therefore, it would be difficult for Young’s IAT to provide comprehensive assessment results in large surveys. Meanwhile, prior large-scale surveys have shown that utilizing clear and succinct instruments is essential for lowering the total number of survey questions, perhaps boosting completion rates, and preventing survey fatigue [31]. The Problematic Internet Use Questionnaire Short Form-6 (PIUQ-SF-6) instrument has six items and was divided into three subdomains: obsession, neglect, and control disorder, which may fulfill the above criteria. Therefore, the current study aimed to determine latent profiles from the PIUQ-SF-6 score, which supports the diagnosis of problematic internet use among a young population with a large sample size. Moreover, acknowledging the gap in data about the associated factors of internet addiction, this study also further explored factors that affect each latent profile of the PIUQ-SF-6 score of Vietnamese youths and adolescents.

## 2. Methods

### 2.1. Study Design, Sampling Method, and Data Collection

A cross-sectional study was performed in Vietnam from January to December 2019. First, according to the purposive sampling method, five provinces/cities (including Tuyen Quang, Ha Noi, Quang Tri, Dak Lak, and Ho Chi Minh City) were selected based on selection criteria, including (i) having both urban and rural populations, and (ii) representing all the north, central, central highlands, and south regions of Vietnam. Second, in each province/city, we randomly selected 320 participants who (1) were youths or adolescents (14–24 years old); (2) were current internet users; and (3) agreed to participate. As a result, 1600 students were recruited to participate in the cyberbullying survey. However, there were 123 people who did not agree to participate, leading to a total of 1477 participants (a response rate of 92.31%) (Table S1).

Respondents were invited to their private rooms for face-to-face interviews and to complete the research questionnaire, which ensured the confidentiality of participant responses. These youths and adolescents, along with their parents or guardians if their age was less than 18, would be provided a written consent form with brief study information. Respondents’ identifiable information was not collected to avoid any social desirability bias as well as facilitate their participation. The protocol of this study was approved by the institutional review board of the Young Research Institute (Code: KXVTN.19-02).

### 2.2. Measurement and Instrument

A structured questionnaire consisting of four major components was developed and used, including:

Internet dependence: We asked participants to respond to the Problematic Internet Use Questionnaire Short Form-6 (PIUQ-SF-6) instrument. This tool had six items and was divided into three subdomains: obsession (2 items), neglect (2 items), and control disorder (2 items). For each item, a 5-point Likert scale ranging from 1—“never” to 5—“almost” was used to evaluate the level of problematic internet use. The total score of six items was calculated, ranging from 6 to 30, with a higher score indicating increased problematic internet use [32]. The Cronbach’s alpha for this instrument was good at 0.873. Besides, participants were requested to report the number of hours spent on the internet.

Socioeconomic characteristic: We asked participants to report a few of their characteristics, including gender (male/female), age, marital status (single/having a partner/being married), living arrangement (family/friend/alone), and living location (urban/rural/mountainous).

Community cohesion: Community cohesion is defined as an individual’s capacity to establish good relationships with their environment and to keep such relations. Development of community cohesion of each individuals depend on their lives and environments [33]. In particular, the ages of youth and adolescence are described as years of social development and cohesion. Eight questions were used to explore participants’ attitudes towards social support and community cohesion around their living areas, including:

- People in this area are willing to help each other.
- People around the living place are willing to help neighbors.
- People live in harmony.
- People around can be trusted and reliable.
- People share the same value and life concept.
- This area has a tremendous amount of trash.
- This area has society’s vices.
- This area has many fights and quarrels.

These questions were divided into 2 domains (neighborhood cohesion [34] with 5 items 1, 2, 3, 4, and 5; neighborhood disorder and social disorder [35] with 3 items 6, 7, and 8). The answer options for each question ranged from 0 (no) to 1 (yes). The total score of these domains was calculated, with a higher score indicating increasing cohesion and disorder. The Cronbach’s alpha for this scale was reported at 0.65.

Health-related variables: Kessler Psychological Distress Scale 6 items (K6) was used to evaluate the psychological distress of participants. This instrument consisted of 6 items to inquire about the participant’s feelings over the past 30 days, including nervous, hopeless, restless/fidgety, depressed, “everything was an effort,” and feelings of worthlessness. A Likert-5 point scale was used for each item, ranging from 0 “None”, 1 “A little”, 2 “Sometimes”, 3 “Most of the time”, and 4 “Always”. The total score of this instrument was summed up, ranging from 0 to 24, with a higher score indicating greater psychological distress [36]. In this study, the Cronbach’s alpha for this scale was good at 0.820. Moreover, we measured self-rated health by employing a visual analogue scale (VAS), with a range score from 0 “The worst health state that you can imagine” to 100 “The best health state that you can imagine” [37].

### 2.3. Statistical Analysis

Latent profile analysis (LPA) is a type of categorical latent variable modeling that aims to identify latent subpopulations within a population based on collected data [38,39,40]. LPA has the potential to answer specific research questions and establish and expand theoretical thinking on the existence of multiple profiles of collected variables [41,42]. In particular, LPA presupposes that, based on the data gathered from participants, each person may be divided into subpopulations with varying degrees of probability. By using LPA, categorical latent variables are identified, and thus investigators can get a parsimonious representation of structures in the form of groups [43]. The PIUQ-SF-6 scale is considered a comprehensive measure to assess the problematic internet use of respondents [32]. But there is a lack of studies that classify this scale into subgroups in Vietnam. Therefore, the LPA was utilized to identify participants having similar responses to problematic internet use in a group of people (categorical latent class).

In this study, MPLUS 8.5 (Asparouhov and Muthén, 2020) [44] software was used to perform latent profile analysis. To carry out LPA, the average scores of the three subscales of PIUQ-SF-6 were used as continuous indicator variables. Models containing one to four latent classes were estimated. To determine the number of latent classes, we used multiple indices: Akaike information criteria (AIC) [45], Bayesian information criteria (BIC) [46], and sample size adjusted Bayesian information criteria (aBIC) [47], with smaller values indicating better model fit, the Lo–Mendell–Rubin likelihood ratio (LMR LR) test, the adjusted LMR LR (aLMR LR) test, and the bootstrap likelihood ratio test (BLRT) [48]. These tests were calculated for model fit decision purposes by comparing models, where a significant *p*-value (*p* < 0.05) showed that a k-class model fits better than the (k-1)-class model [49].

Both descriptive and analytical statistics were performed by STATA version 16. Continuous variables were presented as means and standard deviations (SD), while categorical variables were presented as frequencies with percentages. To compare differences between the level of problematic internet use among participants and other characteristics, Kruskal–Wallis tests were used for continuous variables, and χ^2^ test were utilized for categorical variables. The multinomial logistic regression was conducted to determine factors related to the level of problematic internet use among participants. The potential covariate variables for the full model included socioeconomic characteristics, Kessler score, time using social networking sites, community cohesion characteristics, and visual analogue scale (VAS). A statistically significant *p*-value (P) of 0.05 was used.

## 3. Results

### 3.1. The Characteristics of Problematic Internet Use Questionnaire Short Form

Table 1 describes six items of the PIUQ-SF-6 scale according to three domains. The results are displayed as the percentage of responses and the average score for each question. The majority of participants chose the answer from “Never” to “Sometimes” for all of the questions. On the PIUQ-SF-6 scale, the mean score for each item ranged from 2.1 to 2.7 points.

### 3.2. Comparison of LPA Models with Different Latent Classes Based on Model Selection Statistics

A latent profile class with one to four classes on three domains of PIUQ-SF-6 was estimated, and several selected model fit indices (AIC, BIC, and aBIC) and test values (LMR-LPT, LMRa-LRT, and BLRT) were presented in Table 2. Based on the value of the fit index and tests, the model with three classes was found to be the most suitable. In particular, the values of AIC, BIC, and aBIC decreased significantly when more classes were added to the model, indicating at least two classes were preferred. Additionally, the comparison of the model with two classes and the model with four classes revealed that, although AIC, BIC, and aBIC favored the model with four classes, the examination of three tests (LMR-LPT, LMRa-LRT, and BLRT) showed that the model with four classes should be rejected and favored the 3-class model.

### 3.3. Three Factors of the Problematic Internet Use Questionnaire Short Form Were Obtained from LPA Analysis

**Figure 1:**
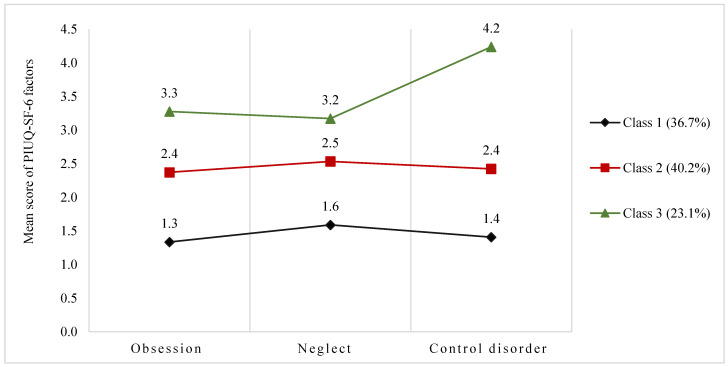
Latent profile analysis on the three factors of the Problematic Internet Use Questionnaire Short Form-6 (*n* = 1477) (Class 1: low risk of problematic Internet use; Class 2: moderate risk of problematic Internet use; Class 3: high risk of problematic internet use.

Figure 1 describes the mean score of three domains of the PIUQ-SF-6 on a 3-class model, which included class 1 “Low risk of Problematic Internet Use”, Class 2 “Moderate risk of Problematic Internet Use”, and Class 3 “High risk of Problematic Internet Use” with 36.7%, 40.2%, and 23.1%, respectively. Compared to classes 2 and 3, the mean score of the three factors of PIUQ-SF-6 in class 1 was the lowest, ranging from 1.3 points to 1.6 points. By contrast, class 3 had the highest mean score of these factors, with 3.2 points in the neglect factor, 3.3 points in the obsession factor, and 4.2 points in the control disorder factor. The average score for class 2 was also reported, ranging from 2.4 to 2.5 points.

### 3.4. Characteristics of Participants According to Three Classes from the Problematic Internet Use Questionnaire Short Form

Table 3 describes the problematic internet use among 1477 participants regarding some characteristics. The majority of participants were female and single, with 62.2% and 81.1%, respectively. The percentage of people at high risk of problematic internet use class living alone (12.6%) was higher than that of people in the moderate-risk class (9.1%) and the low-risk class (3.9%). This difference was statistically significant with *p* < 0.05. Additionally, compared to people in class 1 and class 2, the proportion of people living in urban areas in class 3 was higher than in the other classes. Besides, Hanoi and Tuyen Quang were two cities/provinces where the proportion of people at high risk for problematic internet use was higher than in other provinces such as Quang Tri, Dak Lak, and Ho Chi Minh City. Besides, people who belonged to the lower risk group of problematic internet use had a higher age, Kessler score, and time using the internet than other groups, while the opposite was true for the mean scores of self-rated health, neighborhood cohesion, and neighborhood disorder. These differences were statistically significant with *p* < 0.05.

### 3.5. Selected Results of Multinomial Logistic Regression: Prediction of the Patterns of Problematic Internet Use

Factors related to three patterns of *problematic internet use are presented based on* multinomial logistic regression in Table 4. The higher age was negatively related to the risk of problematic internet use. People with a partner or who are married had a 1.75 times higher odds ratio of being in a high-risk class of problematic internet use (OR = 1.75; 95% CI: 1.20; 2.55; *p* < 0.05) than those at moderate risk. However, the odds ratio of moderate versus low-risk problematic internet use was 0.65 for those who had a partner or were married (OR = 0.65; 95%CI: 0.46; 0.93; *p* < 0.05), indicating that those had 1.54 times higher odds of being in a low-risk class relative to a moderate-risk class of problematic internet use as compared to those who were single.

Regarding living arrangements, compared to people who lived with their family, those who lived alone had a higher odds ratio of being in a high-risk class (OR = 3.47; 95% CI: 1.91; 6.31; *p* < 0.05) and a moderate risk class (OR = 2.66; 95% CI: 1.51; 4.68; *p* < 0.05) relative to the low-risk class of problem internet use. Meanwhile, participants who lived with friends had the odds ratio between high versus moderate risk classes of 0.48 (OR = 0.48; 95% CI = 0.28; 0.81) and the odds ratio between high versus low risk of 0.41 (OR = 0.41; 95% CI = 0.24; 0.71), which indicates that they had 2.08 and 2.43 times higher odds of being in the moderate and low risk classes relative to the high-risk class of problem internet use, as compared to those who lived with family.

Based on Kessler’s score, respondents reporting higher time spent on social networking sites were more likely to belong to moderate and high-risk classes compared to low-risk classes by 1.17 times (OR = 1.17; 95% CI = 1.14; 1.21) and 1.13 times (OR = 1.13; 95% CI = 1.09; 1.18), respectively. Besides, people with a better self-reported health status had a lower likelihood of being in a high-risk class than the remaining two classes.

People living in rural or mountain areas had a lower risk of being in class 3 than in class 2 (OR = 0.62, 95% CI: 0.44; 0.87) or class 1 (OR = 0.68; 95% CI: 0.47; 0.98). In addition, people living in Hanoi City or having higher neighborhood disorder scores were more likely to be at moderate or high risk of problematic Internet use.

## 4. Discussion

This study determined three patterns in internet addiction among Vietnamese youths and adolescents. The majority of respondents were classified as being at moderate or high risk of problematic internet use. Factors such as age, living alone, high Kessler psychological distress score, excessive time on the internet, living in central cities, and having high neighborhood disorder scores were identified as the most associated variables with the risk of internet addiction.

Our study indicated that the high risk of problematic internet use among Vietnamese youths and adolescents was 36.7%, substantially higher than the remaining age groups. This percentage was also high in comparison with the results of other research, such as one conducted in six Asian countries where the highest prevalence of internet addiction came from the Philippines and Hong Kong at 21% and 16%, respectively [50] and another in Vietnam with 20–25% of youths or adolescents addicted to internet use [24,25,26]. The difference in data may be attributed to the gap in sample size. To our knowledge, ours is the largest-scale study with respondents across five main geographical regions (Tuyen Quang, Ha Noi, Quang Tri, Dak Lak, and Ho Chi Minh City). Meanwhile, most of the previous studies were implemented in a local area such as a province/city or university [24,25,26]. Our findings revealed that the area with the highest proportion of participants in the high-risk addictive internet group was Hanoi, while the areas with the lowest proportion of participants in the at-risk group were Quang Tri and Dak Lak. The differences in research regions provided evidence for the hypothesis that internet addiction was associated with access and living conditions. Indeed, the Internet is more accessible and thus more popular in rapidly urbanizing regions or central cities such as Hanoi and Ho Chi Minh City than in suburban or rural/mountainous areas such as Quang Tri and Dak Lak. This difference could also be attributed to a disparity in community cohesion characteristics, as the stability level of the neighborhood environment in Hanoi was lower than that in Ho Chi Minh City. Two of the above-mentioned factors are individual and neighborhood characteristics and will therefore be further discussed in this study.

Regarding individual characteristics, factors related to the moderate and high risk of internet addiction profiles included age, living alone, a higher Kessler score, and excessive time on the internet. The first predictor of internet addiction is age, meaning participants are more likely to develop moderate- or high-risk profiles as their age increases. The correlation between age and an increasing risk of internet abuse was also indicated in two studies in Hong Kong, where older adolescents displayed more problematic internet use [51,52]. This could be explained by the complex dynamics of an older adolescent’s development. Compared to younger children, older adolescents have a greater need to establish a sense of identity on the internet [52]. Moreover, older adolescents tend to have more freedom in the utilization of smartphone devices, while younger teenagers’ daily internet usage is often monitored by parents. The second predictor of internet addiction is living status. Our results suggested that people living alone were more likely to be subject to a moderate or high risk of problematic internet use. The majority of people who live alone, especially young people or adolescents, usually feel lonely. Most previous studies have also demonstrated that loneliness is an important factor associated with internet addiction [53,54]. For instance, in Greece, the rates of internet addiction in adolescents grew exponentially, reaching the rates of loneliness in internet-addicted students [55]. This trend may be explained by the fact that the internet and online platforms in general are convenient means of entertainment that do not require the participation of other people, and therefore, adolescents living alone are generally more attracted to the internet and more susceptible to internet abuse. In our study, a high Kessler psychological distress score was also a significant factor in the risk of internet addiction, and a wealth of existing literature also proved it [56,57]. As the perception of internet dependence has been leaning towards a “psychopathological disorder” [58,59], it was hypothesized that internet addiction cases usually appear under the same trends as impulse control or addictive symptoms [60], with its own potential biomarkers being identified [56]. Despite ongoing research attention, the bidirectional relationship between mental health issues and internet addiction has not been elucidated, and further longitudinal studies are needed to determine the mutual interaction between the above variables. Excessive time on the internet was the last associated individual factor investigated in this study. According to Chak and Leung (2014), the amount of time internet addicts spent online accumulated to an extra 1.08 days per week compared to their peers [61]. This alarming pattern of internet use came with its consequences: a significant increase in internet usage was often accompanied by a loss of awareness of time or disregard of daily tasks and withdrawal symptoms in the absence of the internet [62,63]. In Vietnam, previous researchers have determined consequences of internet abuse in the field of psychological and functional disorders such as sleep-related disorders [27], anxiety, depression, discomfort, and overall well-being such as pain, difficulty in performing self-care tasks, daily routine, and poor health-related quality of life in youths and adolescents [25].

Regarding social and neighborhood characteristics, people residing in urban areas and having more neighborhood disorders were more likely to fall into high-risk groups for problematic internet use. This finding suggested that a safe living environment with fewer social vices had positive effects on reducing internet dependence among teenagers, meaning those in areas with high community cohesion tended to be less dependent on the internet. In contrast, those in hazardous neighborhoods were more likely to be addicted to the internet. This finding aligned with existing studies, in which it was implied that in safe and pollution-free environments, people are more likely to engage in outdoor and physical activities, which reduce their internet usage [64,65,66]. Studies in other developed countries such as Australia, New Zealand, and the USA also indicated a positive association between stable neighborhoods and appropriate use of the internet among youths [67,68,69,70]. Moreover, people are more likely to develop stress in neighborhoods with high levels of social disorder [71]. Numerous research documented the association between high stress and psychological factors including loss of control, inappropriate behavior and inability to restrain from impulses, all of which were underlying causes of Internet abuse [15,72]. Residents of socially disorganized neighborhoods have low adaptability to stress and tend to avoid stress or withdraw from social life altogether as a coping mechanism. Given the surge in stress levels among youths and adolescents in recent years, this pattern is highly dangerous for their well-being and psychological state. In response to stress, youths might develop a virtual life through mediated social networks and limit real-life communications [73].

Several interventions can be inferred from this study’s findings. Firstly, as internet addiction leads to poorer health status, especially mental health, frequent screening should be provided for teenagers. These activities can be implemented at school/university/college or in living places with the support of schools, parents, family members, and social organizations [74]. Likewise, early intervention training on restricting internet use should be developed to provide youths and adolescents, as well as their parents/family members, with an understanding of internet harm and encourage them to engage in healthier activities such as sports, arts, etc. [75]. Moreover, since neighborhood characteristics have direct impacts on internet dependence, policymakers should consider interventions on an interpersonal level such as improving living conditions with more social cohesion and higher security for adolescents. These measures promote face-to-face relationships and encourage young people to engage more in outdoor activities [76,77,78]. In addition, it is necessary to identify high-risk sites of internet addiction, such as large cities and urban areas with dense populations, in order to develop appropriate intervention strategies. Finally, longitudinal studies should be conducted on hypothesized variables associated with internet addiction.

There are several limitations to our study. First, due to the nature of cross-sectional studies, the causal relationship could not be determined, and self-reported data might lead to recall bias. Secondly, participants might not be representative of the youth and adolescents, as we could only investigate 5 out of 63 provinces in Vietnam. However, data were collected with different geographic and cultural characteristics to mitigate this limitation. Thirdly, participant characteristics skewed mainly towards single adolescent females living with their families. Hence, this might be limited relatively to the generalizability of the findings, although it does not invalidate the findings. Lastly, there might be a social desirability bias that appears when this study is related to social science research. Nevertheless, this study represents the largest effort to date in measuring internet addiction through its association with individual and neighborhood characteristics in Vietnam.

## 5. Conclusions

Our study underlined that using the latent profile seems to be an appropriate measure to differentiate between problematic Internet users when this problem has not yet become a gold standard for classification. Individual factors such as age, living alone, higher levels of psychological distress, excessive time on the Internet, and neighborhood characteristics such as living in central cities and high neighborhood disorder scores were found to be related to moderate and high risks of internet addiction among youths and adolescents. This study informs early interventions for decreasing internet addiction among Vietnamese youths, including raising awareness, encouraging outdoor activities, implementing psychology therapy, improving living conditions, identifying high-risk sites, as well as developing youth-oriented policies.

## Figures and Tables

**Table 1:** The characteristics of Problematic Internet Use Questionnaire Short Form-6.

Factors	Items	Respond (%)					Mean	SD
		Never	Rarely	Sometime	Often	Always		
Obsession	How often do you feel tense, irritated, or stressed if you cannot use the Internet for as long as you want to?	36.3	32.1	23.0	5.9	2.8	2.1	1.0
	How often does it happen to you that you feel depressed, moody, or nervous when you are not on the Internet and these feelings stop once you are back online?	27.8	20.0	23.2	12.2	16.8	2.7	1.4
Neglect	How often do you spend time online when you’d rather sleep?	26.1	36.2	28.9	8.5	0.3	2.2	0.9
	How often do people in your life complain about spending excessive time on internet use?	32.4	24.0	22.8	12.3	8.6	2.4	1.3
Control disorder	How often does it happen to you that you wish to decrease the amount of time spent online but you do not succeed?	36.5	27.3	15.6	13.4	7.2	2.3	1.3
	How often do you try to conceal the amount of time spent online?	45.3	21.1	13.1	10.4	10.2	2.2	1.4

**Table 2:** Comparison of LPA models with different latent classes based on model selection statistics. Criteria to assess model fit for latent profile analysis models.

Number Latent Class	AIC	BIC	aBIC	LMR-LRT (*p*-Value)	LMRa-LRT (*p*-Value)	BLRT (*p*-Value)
Class 1	12,831.71	12,863.49	12,844.43	-	-	-
Class 2	11,198.81	11,251.79	11,220.02	<0.01	<0.01	<0.01
Class 3 ***	10,854.80	10,928.97	10,884.49	<0.01	<0.01	<0.01
Class 4	10,731.65	10,827.01	10,769.83	0.23	0.24	<0.01

***: *Optimal number of latent classes*.

**Table 3:** Characteristics of participants according to three classes of problematic internet use.

Questionnaire Short Form									
Characteristics	Risk of Problematic Internet Use						Total		*p*-Value
	Class 1 (Low Risk)		Class 2 (Moderate Risk)		Class 3 (High Risk)				
	*n*	%	*n*	%	*n*	%	*n*	%	
Total	542	36.7	594	40.2	341	23.1	1477	100.0	
Gender									
Male	231	42.6	206	34.7	116	34.0	553	37.4	0.007
Female	311	57.4	388	65.3	225	66.0	924	62.6	
Marital status									
Single	432	79.7	512	86.2	264	77.4	1208	81.8	<0.01
Having partner/being married	110	20.3	82	13.8	77	22.6	269	18.2	
Living arrangement									
Family	453	83.6	469	79.0	274	80.4	1196	81.0	<0.01
Friends	68	12.5	71	12.0	24	7.0	163	11.0	
Alone	21	3.9	54	9.1	43	12.6	118	8.0	
Location									
Urban	332	61.3	335	56.4	255	74.8	922	62.4	<0.01
Rural/mountain areas	210	38.7	259	43.6	86	25.2	555	37.6	
Province									
Tuyen Quang	96	17.7	117	19.7	79	23.2	292	19.8	<0.01
Ha Noi	68	12.5	98	16.5	106	31.1	272	18.5	
Quang Tri	128	23.6	137	23.1	55	16.1	320	21.7	
Dak Lak	132	24.4	148	24.9	40	11.7	320	21.7	
Ho Chi Minh City	118	21.8	94	15.8	61	17.9	273	18.5	
	Mean	SD	Mean	SD	Mean	SD	Mean	SD	*p*-Value
Age, years	18.5	2.0	19.0	2.1	19.0	2.3	18.8	2.1	<0.01
Kessler score (0–24)	3.9	3.9	7.3	4.7	6.3	4.5	5.8	4.6	<0.01
Time using social network sites per day (hours)	3.8	2.6	4.6	3.3	3.9	3.0	4.1	3.0	<0.01
Visual analogue scale (VAS) (0–100)	89.0	12.8	82.5	15.1	81.3	17.5	84.6	15.3	<0.01
Community cohesion									
Neighborhood cohesion (0–5)	2.3	1.5	2.3	1.5	2.5	1.4	2.4	1.5	0.060
Neighborhood disorder (0–3)	0.1	0.4	0.3	0.5	0.5	0.8	0.3	0.6	<0.01

**Table 4:** Selected results of multinomial logistic regression: prediction of the patterns of problematic internet use.

	Risk of Problematic Internet Use					
	High vs. Moderate Risk		Moderate vs. Low Risk		High vs. Low Risk	
	OR	95% CI	OR	95% CI	OR	95% CI
Individual Characteristics						
Age (per year)	1.06	0.98; 1.14	1.12 ***	1.04; 1.21	1.18 ***	1.09; 1.29
Gender (female vs. male ref)	1.16	0.86; 1.57	1.15	0.88; 1.51	1.34 *	0.97; 1.84
Marital status (single ref)						
Having partner/being married	1.75 ***	1.20; 2.55	0.65 **	0.46; 0.93	1.14	0.78; 1.67
Living arrangement (family ref)						
Friends	0.48 ***	0.28; 0.81	0.86	0.57; 1.29	0.41 ***	0.24; 0.71
Alone	1.31	0.82; 2.09	2.66 ***	1.51; 4.68	3.47 ***	1.91; 6.31
Time using social network sites (per hours)	0.95 **	0.90; 1.00	1.10 ***	1.05; 1.15	1.04	0.98; 1.10
Kessler score (per score)	0.96 **	0.93; 1.00	1.17 ***	1.14; 1.21	1.13 ***	1.09; 1.18
Self-reported health status (per score)	1.00	0.99; 1.00	0.98 ***	0.97; 0.99	0.98 ***	0.97; 0.99
Community Characteristics						
Province (Tuyen Quang ref)						
Ha Noi	2.09 ***	1.28; 3.41	2.30 ***	1.40; 3.79	4.82 ***	2.80; 8.28
Quang Tri	0.85	0.53; 1.37	1.18	0.77; 1.81	1.00	0.60; 1.67
Dak Lak	0.61 *	0.36; 1.03	0.99	0.64; 1.55	0.61 *	0.35; 1.06
Ho Chi Minh City	1.11	0.68; 1.80	1.15	0.74; 1.79	1.27	0.77; 2.10
Location (rural/mountainous vs. urban ref)	0.62 ***	0.44; 0.87	1.10	0.82; 1.48	0.68 **	0.47; 0.98
Community cohesion						
Neighborhood cohesion (per score)	1.08	0.98; 1.19	1.06	0.97; 1.16	1.14 **	1.03; 1.27
Neighborhood disorder (per score)	1.70 ***	1.37; 2.11	1.46 ***	1.12; 1.92	2.49 ***	1.89; 3.28

*** *p* < 0.01, ** *p* < 0.05, * *p* < 0.1.

## Data Availability

Data are available upon request due to privacy restrictions.

## Supplementary Materials

The following supporting information can be downloaded at: https://www.mdpi.com/article/10.3390/ijerph20032090/s1, Table S1: The characteristics of participants regarding five provinces.
