# Supplementary material for: Association of Individual and Neighborhood Characteristics to Problematic Internet Use among Youths and Adolescents: Evidence from Vietnam
Source: Int J Environ Res Public Health. 2023 Jan 23;20(3):2090. doi: 10.3390/ijerph20032090 (PMC9915430; doi:10.3390/ijerph20032090)
Supplement: Supplementary file 1 [file ijerph-20-02090-s001.zip › ijerph-1996934-supplementary.pdf]

Table S1. The characteristics of participants regarding five provinces.

	Tuyen Quang		Hanoi		Quang Tri		Dak Lak		Ho Chi Minh city		*p*-Value
	n	%	n	%	n	%	n	%	n	%	
**Gender (Female)**	188	64.4	154	56.6	192	60.0	239	74.7	151	55.3	<0.01
**Marital status**											
Single	233	79.8	231	84.9	245	76.6	316	98.8	183	67	<0.01
Having partner/being married	59	20.2	41	15.1	75	23.4	4	1.3	90	33	
**Location**											
Urban	218	74.7	226	83.1	113	35.3	125	39.1	240	87.9	<0.01
Rural/mountain areas	74	25.3	46	16.9	207	64.7	195	60.9	33	12.1	
**Living arrangement**											
Family	260	89	206	75.7	236	73.8	281	87.8	213	78	<0.01
Friends	16	5.5	37	13.6	44	13.8	24	7.5	42	15.4	
Alone	16	5.5	29	10.7	40	12.5	15	4.7	18	6.6	
	**Mean**	**SD**	**Mean**	**SD**	**Mean**	**SD**	**Mean**	**SD**	**Mean**	**SD**	**p-value**
**Age, years**	20.0	2.4	17.5	1.6	19.6	1.5	18.1	1.5	18.8	2.4	0.042
**Time using social network sites per day (hours)**	3.9	2.7	3.4	2.5	3.9	3.0	4.6	3.4	4.8	3.2	<0.01
**Kessler score (0-24)**	6.9	4.4	5.5	4.1	5.6	5.0	6.7	4.9	4.5	4.1	<0.01
**Community cohesion**											
Neighborhood cohesion (0-5)	3.0	1.4	2.1	1.4	2.2	1.5	2.2	1.5	2.3	1.5	<0.01
Neighborhood disorder (0-3)	0.3	0.7	0.3	0.6	0.2	0.5	0.3	0.7	0.2	0.5	0.056
